# The journey of an electrifying (r)evolution

**DOI:** 10.1038/s41467-021-24410-3

**Published:** 2021-06-30

## Abstract

From smartphones to electric vehicles, Li-ion batteries have revolutionized our daily lives. Here, we discuss the most important aspects that have enabled lithium-ion batteries to thrive, and introduce some of our articles that contribute to the evolution of these devices.

During the 1960s and ’70s, the oil crisis inspired research towards innovative electrochemical energy storage technologies that could reversibly store much larger energies than those already available in, e.g., lead-acid or Ni-Zn batteries^1^.

In this context, the discoveries made by the 2019 Chemistry Nobel laureates M. Stanley Whittingham, John B. Goodenough and Akira Yoshino^2^ enabled the fast development of rechargeable batteries that ultimately culminated in the commercialization of the first Li-ion rechargeable battery in 1991^3^.

A Li-ion battery’s electrochemically active components (e.g., cathode and anode) are the core of this energy storage system. Concerning the cathode, Whittingham’s discovery in the late 70s of a layered transition metal sulphide (i.e., TiS_2_) able to store charge at potentials >2 V vs Li/Li^+^ opened new scientific research pathways in the battery field^4^. However, this material still had a significant limitation: the potential of TiS_2_ was not sufficient to produce a high-energy battery. To tackle this drawback, Goodenough proposed a layered transition metal oxide (i.e., LiCoO_2_) that grants a cell voltage of about 4 V^5^.

Nevertheless, as for many energy storage devices, stability remained an issue. Indeed, the anode used in the early days of battery research was lithium metal that has proven to be unstable upon prolonged cycling due to the formation of dendritic structures, which eventually lead to cell short circuiting^5^. To circumvent the issue of Li metal anode, Yoshino proposed using a carbonaceous material to store Li ions at low potentials (i.e., <0.5 V vs Li/Li^+^), which resulted in a non-excessive energy loss and enhanced safety compared to lithium metal^3^.

LiCoO_2_ and carbon materials gave birth to the first generation of Li-ion batteries in the early ’90s. In the following years, other Li-ion battery designs with greater energy storage and longer lifetimes emerged. These energy storage devices triggered many technological wonders over the last few decades and today, the latest generations of Li-ion batteries are ubiquitous in our daily lives.

However, the design of new batteries is not over yet^6^. In the prior two decades, scientists have devoted tremendous efforts to investigate a massive variety of materials to fully exploit and enhance the Li-ion battery system’s charge storage performances. New cathode materials^6^, solid electrolyte implementation, innovative characterizations methods^6,7^, advanced cell concepts (e.g., anode-free cell), and novel Li metal anode stabilization strategies are only some of the significant approaches proposed by the researchers to produce high-energy Li-based cells.

To showcase the recent developments in this vibrant field, we are delighted to unveil a collection of some of the most exciting articles published in *Nature Communications*, which reports cutting-edge research works in the fields of cathode active materials, metal anodes and solid electrolytes.

The incredible journey of this electrifying (r)evolution continues. In the years to come, we are confident that many young scientists will contribute to the research, discovery, and development of new energy storage systems capable of sustaining future human societies in the Anthropocene era.
